# Missed Opportunities to Address Cardiovascular Disease Risk Factors amongst Adults Attending an Urban HIV Clinic in South Africa

**DOI:** 10.1371/journal.pone.0140298

**Published:** 2015-10-08

**Authors:** Miriam Rabkin, Anthony Mutiti, Christine Chung, Yuan Zhang, Ying Wei, Wafaa M. El-Sadr

**Affiliations:** 1 ICAP at Columbia, Columbia University Mailman School of Public Health, New York, New York, United States of America; 2 Department of Epidemiology, Columbia University Mailman School of Public Health, New York, New York, United States of America; 3 Department of Medicine, Columbia University College of Physicians and Surgeons, New York, New York, United States of America; 4 ICAP in South Africa, Pretoria, South Africa; 5 ICAP in Malawi, Lilongwe, Malawi; 6 Department of Biostatistics, Columbia University Mailman School of Public Health, New York, New York, United States of America; Azienda ospedaliero-universitaria di Perugia, ITALY

## Abstract

We assessed cardiovascular disease (CVD) risk factor prevalence and risk stratification amongst adults on antiretroviral therapy in South Africa. Of the 175 patients screened, 37.8% had high blood pressure (HBP), 15.4% were current smokers, 10.4% had elevated cholesterol, and 4.1% had diabetes, but very few (3.6%) had a 10-year CVD risk >10%. One-third of those with HBP, 40% of those with diabetes, and two-thirds of those with high cholesterol had not previously been diagnosed. Although participants were adherent with chronic HIV care, screening for and management of CVDRF were suboptimal, representing a missed opportunity to reduce non-AIDS morbidity and mortality.

## Introduction

Although HIV remains the leading cause of death among adults in sub-Saharan Africa, the burden of non-communicable disease (NCD) is high and rising rapidly [1]. In many African countries, cardiovascular disease (CVD) is a leading cause of death [2,3], and the prevalence of CVD risk factors (CVDRF) such as diabetes, dyslipidemia, hypertension, and tobacco use is significant [4,5,6]. South Africa, for example, is tackling one of the world’s most severe HIV epidemics, while also confronting a growing CVD burden [7]. CVD causes 18% of deaths in the country, and CVDRF have been described as a “time bomb” for South Africa’s health system [8,9]. This convergence of HIV and NCDs presents both challenges and opportunities for public health.

The scale-up of HIV care and treatment has led to growing survival and longevity of people living with HIV (PLWH), who are increasingly at risk for the NCDs prevalent in their communities. Data from sub-Saharan Africa are limited, but PLWH appear to be at higher risk than the general population for CVD and CVDRF [10,11,12]; dyslipidemia and diabetes are also associated with some antiretroviral drugs [13]. As PLWH age on antiretroviral therapy (ART), determining the best approaches for integrating HIV services and the management of common CVDRF will be critical to maintain the advances of treatment scale-up, and to assure optimal overall health outcomes.

Developing strategies that effectively reduce CVD risk, optimize resource utilization, and do not undermine HIV-related program and patient outcomes is an important challenge [14]. In order to be effective, interventions must be contextually appropriate, feasible for low-resource settings, and cognizant of limited human resources and fragile health systems.

This study assessed the prevalence of CVDRF among adults on ART at an urban HIV clinic in South Africa and explored the feasibility of using CVD risk stratification to streamline CVDRF management.

## Methods

### Study setting and participants

The study was conducted at an urban Community Health Centre (CHC) in South Africa’s Free State Province. The CHC serves an urban population and provides approximately 3,000 outpatient visits a month; its HIV clinic has enrolled 1,900 adults, 65% of whom are female. Nurses and counselors typically manage HIV patients, although a physician is available one day per week for consultation on complex cases.

The study recruited a convenience sample of adult PLWH from the HIV clinic within the CHC. Patients were eligible if they were 30 years or older, had been receiving ART for at least one year, had access to a mobile phone, and were not acutely ill, pregnant, or breastfeeding. Eligible patients were informed of the study by their HIV clinicians; those interested in participating were referred to study staff for screening. The study was explained, as was the right to decline participation, and informed consent was obtained from interested patients.

Ethical approvals were provided by the Institutional Review Boards at Columbia University (IRB-AAAM4408) and the University of the Free State (143/2013). Participants provided written informed consent to participate in the study.

### Data collection procedures

Survey and consent forms were developed in English, translated into Sesotho, back-translated into English, revised, and then piloted in Sesotho for clarity. Trained bilingual study nurses administered the survey in the participant’s choice of Sesotho or English; the survey took approximately 90 minutes and included questions on demographics, household characteristics, health status, family history, and CVDRF. Questions regarding physical activity were excerpted from the International Physical Activity Questionnaire (IPAQ) short form [15,16]. Questions regarding diet were adapted from the WHO STEPS survey instrument [17]. The study nurses used structured chart abstraction forms to collect data on participant medical history.

Weight was measured using calibrated digital scales, with shoes and outwear removed. Height was measured using a calibrated stadiometer. Sitting blood pressure (BP) was measured using a digital BP cuff; two measurements were taken at least five minutes apart. Blood was collected via phlebotomy and sent to an accredited local laboratory.

Results of the survey, physical examination, chart abstraction and laboratory testing were entered onto paper-based case report forms. Forms were reviewed for completeness and accuracy, and data were entered into SAS version 9.4 (SAS Institute, Cary, North Carolina).

### Measures

The primary outcome measures were the prevalence of four CVDRF (hypertension, high cholesterol, diabetes, and tobacco smoking) and participants’ 10-year risk of a cardiovascular event, as defined by the WHO/ISH risk stratification tool [18,19]. For hypertension, the average of the first and second BP measurement was used and participants were categorized as having high blood pressure if the average systolic BP was greater than or equal to 140 mmHg and/or if the average diastolic BP was greater than or equal to 90 mmHg, as per standard staging guidelines [20]. Diabetes was defined as HbA1c measurement above 6.5% and high cholesterol was defined as a random total cholesterol measurement above 240 mg/dL. Active tobacco smoking was defined as self-reported cigarette, pipe, or cigar smoking within the past 30 days. Overweight was defined as a body mass index (BMI) of 25.0–29.9 and obese was defined as a BMI of 30.0 or greater.

Ten-year risk of a cardiovascular event was defined using the WHO/ISH risk stratification charts, which use six variables (gender, age, systolic blood pressure, total cholesterol, smoking and presence/absence of diabetes) to stratify the ten-year absolute risk of stroke and heart attack in adults aged 40–79 into five risk categories. The WHO/ISH risk charts are based on modeling using regional population-level data on the relative and absolute risk of each risk factor [21].

### Statistical analysis

Data were imported into SAS 9.4 for analysis. Descriptive statistics including means/proportions and their standard deviation were calculated for all variables of interest. Due to small expected cell counts, Fisher’s exact tests were used to determine whether the differences in current tobacco use, high cholesterol, diabetes, underweight and obesity were statistically significant between male and female at significant level of 0.05.

## Results

Between March and June of 2014, complete data were collected for 175 participants, of whom 130 (74.2%) were female. Average age was 45.4 years (SD ± 8.8). Table 1 shows participant demographics, medical history, and CVDRF prevalence. On average, participants had been attending the HIV clinic for four years, and their mean CD4^+^ count was 411/mm^3^ (SD±184). Of the 143 participants with a documented viral load, 65.7% had undetectable levels. Most participants (82.2%) were taking ART regimens that included a nucleoside analog backbone of lamivudine and tenofovir, and (90.9%) were taking nevirapine or efavirenz; none were on protease inhibitors. Only 8% of participants reported missing a dose of ART in the past month, and only 5.1% reported missing an appointment at the HIV clinic within the past three months.

**Table 1:** Participant Demographics, Health History, and CVD RF Prevalence.

		All (N = 175)	Female (N = 130)	Male(N = 45)
**Demographic and Socioeconomic**	* *			
Sex, female		130 (74.2%)		
Age, mean (SD)		45.4 (8.8)	44.7 (9.0)	47.6 (7.9)
Population group	*Black/African*	173 (98.9%)	128 (98.5%)	45 (100%)
Marital status	*Currently married or living with partner*	56 (32.0%)	34 (26.2%)	22 (48.9%)
Educational attainment	*Any secondary*	66 (37.9%)	41 (31.5%)	25 (55.6%)
	*Completed secondary*	75 (43.1%)	61 (46.9%)	14 (31.1%)
Household has electricity		158 (90.3%)	121 (93.1%)	37 (82.2%)
Household has piped water		175 (100%)	130 (100%)	45 (100%)
**Health—HIV history**	* *			
Most recent CD4, mean (SD)		411 (184)	395 (213)	322 (175)
Most recent viral load	*missing*	32 (18.9%)	30 (23.1%)	2 (4.4%)
	*undetectable*	94/143 (65.7%)	67/100 (67.0%)	27/45 (60.0%)
	*detectable*	49/143 (34.3%)	33/100 (33.0%)	16/45 (40.0%)
	*mean (SD) for those with detectable results*	8,693 (26,454)	4,326 (9,028)	17,700 (44,012)
On nevirapine or efavirenz		159 (90.9%)	119 (90%)	42 (93.3%)
On protease inhibitor-containing regimen		0	0	0
**Health—NCD history**				
Prior diagnosis of hypertension (HTN) documented in chart		52 (29.9%)	46 (35.6%)	6 (13.3%)
Prior diagnosis of diabetes (DM) documented in chart		7 (4%)	5 (3.9%)	2 (4.4%)
Prior diagnosis of high cholesterol/hyperlipidemia documented in chart		14 (8%)	13 (10.1%)	1 (2.2%)
Prior documentation of cigarette smoking (ever documented)		1 (0.6%)	0	1 (2.2%)
Prior documentation of weight (ever documented)		173 (99.4%)	129 (100%)	44 (97.8%)
Prior diagnosis of obesity documented in chart		0	0	0
High BP documented at most recent visit	Any high BP (SBP *>*140 and/or DBP ≥ 90)	61 (34.9%)	46 (35.4%)	15 (33.3%)
	*Grade 1 (SBP 140–159 and/or DBP 90–99)*	41 (23.4%)	32 (24.6%)	9 (20%)
	*Grade 2 (SBP* *≥* *160 and/or DBP* *≥* *100)*	20 (11.4%)	14 (10.8%)	6 (13.3%)
Of pts with diagnosis of HTN, percent with high BP when last documented		63.5%	61%	83%
**Awareness of CVDRF (self-report)**				
Told of DM by health provider		14 (8%)	12 (9.2%)	2 (4.4%)
Told of HTN by health provider		56 (32%)	49 (37.7%)	7 (15.6%)
Told of high cholesterol by health provider		5 (2.9%)	4 (3.1%)	1 (2.2%)
Told of “heart disease” by health provider		11 (6.3%)	10 (7.7%)	1 (2.2%)
Told of obesity or overweight by health provider		19 (10.9%)	17 (13.1%)	2 (4.4%)
**Findings at Study Visit**				
High BP	Any high BP (SBP *>*140 and/or DBP ≥ 90)	66 (37.7%)	50 (38.5%)	16 (35.6%)
	*Grade 1 (SBP 140–159 and/or DBP 90–99)*	41 (23.4%)	31 (23.8%)	10 (22.2%)
	*Grade 2 (SBP* *≥* *160 and/or DBP* *≥* *100)*	25 (14.3%)	19 (14.6%)	6 (13.3%)
BMI	*Underweight (BMI <18*.*5)*	17 (9.8%)	8 (6.2%)	9 (20%)
	*Overweight (BMI 25–29*.*0)*	37 (21.1%)	29 (22.3%)	8 (17.8%)
	*Obese (BMI* *≥* *30)*	50 (28.6%)	48 (36.9%)	2 (4.4%)
HbA1c	*HbA1c > 6*.*5%*	7 (4.1%)	6 (4.7%)	1 (2.2%)
Total Cholesterol, mean (SD) in mg/dL	*mean TC*	184.8 (41.38)	188.2 (40.85)	175.4 (41.65)
	*TC > 240 mg/dL*	18 (10.4%)	15 (11.7%)	3 (6.6%)
Ever smoked tobacco products (cigarette, cigar, pipe)?		54 (30.9%)	16 (12.3%)	38 (84.4%)
Currently smokes tobacco products (in past 30 days)		27 (15.4%)	4 (3.1%)	23 (51.1%)
Currently smokes tobacco daily		23 (13%)	3 (2.3%)	20 (44.4%)

At the time of the study visit, 37.7% of participants had high blood pressure (HBP). Of those with HBP, 64.9% had a documented prior diagnosis of hypertension, and their previous BP control had been poor, with nearly two-thirds of these participants (63.5%) having documented HBP at their most recent HIV clinic visit. More than half of participants (52.5%) reported an immediate family member with hypertension.

Tobacco smoking had been documented in less than 1% of charts, however when interviewed, 30.9% of participants reported a prior history of tobacco use and 15.4% were current tobacco smokers (3.1% women, 51.1% men, p < 0.001). More than half (53.1%) of smokers had attempted to quit within the past year.

10.4% of participants had random total cholesterol > 240 mg/dL; hyperlipidemia was previously documented in less than a third (27.9%) of these patients. Diabetes was found in seven patients (4.1%); it had been previously documented in only four of the seven (57%). Notably, although self-reported activity levels were relatively high, 21.1% of participants were overweight (22.3% women, 17.8% men) and 28.6% were obese (36.9% women, 2.0% men, p < 0.001). Almost all participants (99.4%) had at least one documented weight in their clinic chart, although only 10.9% reported being told of obesity or overweight by their clinicians.

**Fig 1:**
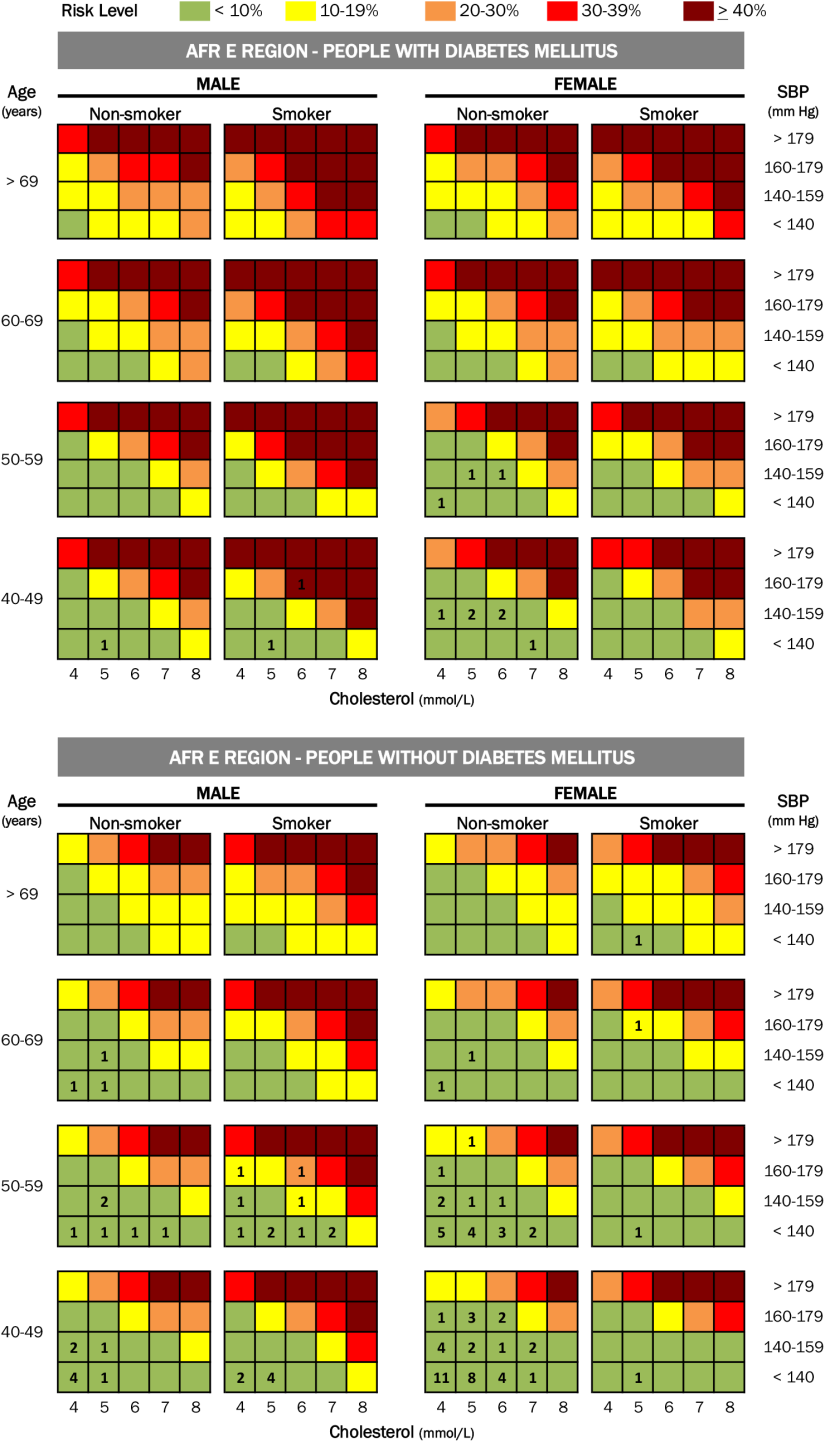
shows the results for the 110 participants eligible for stratification, superimposed on the WHO/ISH risk stratification charts for the AFR-E region.

CVD risk was stratified for the 110 participants (76 women, 36 men) who were 40 years and older. Almost all (96.4%) had a 10-year CVD risk of less than 10%. Two (1.8%) had CVD risk of 10–19%, one (0.9%) had CVD risk of 20–29% and one (0.9%) had CVD risk of ≥ 40%. Fig 1 shows risk stratification for the entire group.

## Discussion

CVDRF were common amongst PLWH on ART at this urban HIV clinic, as they are in the general population of Free State Province [22]. Rates of overweight and obesity were also similar to those reported for the general population of Free State, where estimated rates are 19.5% and 5.8% respectively amongst men and 20.7% and 43% respectively amongst women [22]. Compared to other studies of South Africa PLWH on ART, the rate of diabetes was similar [23] and the rate of HBP was somewhat higher [24,25], likely due to the older age of the study participants. Few studies have described rates of tobacco smoking or high total cholesterol amongst comparable cohorts in South Africa.

Although the participants in this study were enrolled in and adherent with longitudinal chronic care, visiting the HIV clinic every one to three months for years, one-third of those with HBP, 40% of those with diabetes, and two-thirds of those with high cholesterol had not previously been diagnosed with these conditions. Overweight, obesity, and tobacco smoking were rarely documented in clinic charts, and few patients reported receiving behavioral counseling regarding weight loss and smoking cessation. Hypertension was poorly controlled, with 34.9% of participants having HBP at their most recent clinic visit and 37.7% found to have HBP at the study visit. The fact that participants were likely somewhat older, retained in care for longer, and more adherent to treatment than other PLWH suggests that they might be *more* likely to have been screened and treated for CVDRF than other patients. The fact that even these patients had not been adequately screened for CVDRF and managed appropriately suggests that there may be an important gap in these services.

CVD risk stratification is useful in guiding the intensity of CVDRF management and in prioritizing high-risk individuals [26,27]. In this study, almost all participants fell into the same risk category, limiting the use of WHO/ISH risk charts for triage purposes. The fact that 96.4% of participants 40 years and older had less than 10% ten-year risk of a cardiovascular event suggests that initial management of CVDRF within HIV clinic, rather than immediate referral to specialists, may be an effective approach.

Little has been published regarding optimal approaches to screening PLWH for CVDRF in low-resource settings, and this study adds to the scant literature on the topic. This study has some limitations, notably its focus on a single urban HIV clinic and its non-random sampling, both of which limit generalizability. Blood pressure was measured during a single visit, rather than on different days as suggested by clinical guidelines. However, this approach has been utilized widely in comparable studies, and in the 2013 South Africa National Health and Nutrition Survey (SANHANES-1) [28,29]. Nonfasting total cholesterol was used to diagnose hypercholesterolemia, which may therefore have been overestimated.

The results of this study have implications for policy-makers and program directors, indicating a substantial missed opportunity to identify and treat CVDRF amongst PLWH enrolled in continuity care for HIV. As CVDRF become increasingly common amongst both PLWH and the general population, health systems must confront both HIV and CVD risk factors if the successes of HIV scale up are to be maintained.

## Supporting Information

S1 Table Demographic information, assets, and HIV history. (XLSX)

S2 Table Prevalence of CVD risk factors (self report, chart review and study visit). (XLSX)
